# Oseltamivir for coronavirus illness: post-hoc exploratory analysis of an open-label, pragmatic, randomised controlled trial in European primary care from 2016 to 2018

**DOI:** 10.3399/bjgp20X711941

**Published:** 2020-06-23

**Authors:** Samuel Coenen, Alike W van der Velden, Daniela Cianci, Herman Goossens, Emily Bongard, Benjamin R Saville, Nina Gobat, Muireann de Paor, Margareta Ieven, Theo J Verheij, Christopher C Butler

**Affiliations:** Centre for General Practice, Department of Family Medicine & Health Policy (FAMPOP); Laboratory of Medical Microbiology, Vaccine & Infectious Disease Institute (VAXINFECTIO), University of Antwerp, Antwerp, Belgium.; Julius Center for Health Sciences and Primary Care, University Medical Center Utrecht, Utrecht University, Utrecht, the Netherlands.; Julius Center for Health Sciences and Primary Care, University Medical Center Utrecht, Utrecht University, Utrecht, the Netherlands.; Laboratory of Medical Microbiology, Vaccine and Infectious Disease Institute (VAXINFECTIO), University of Antwerp, Antwerp; Laboratory of Clinical Microbiology, Antwerp University Hospital, Edegem, Belgium.; Department of Primary Care Health Sciences, University of Oxford, Radcliffe Observatory Quarter, Oxford, UK.; Berry Consultants, Austin, Texas, US; adjunct assistant professor, Vanderbilt University, Department of Biostatistics, Nashville, Tennessee, US.; Department of Primary Care Health Sciences, University of Oxford, Radcliffe Observatory Quarter, Oxford, UK.; Department of General Practice, Royal College of Surgeons in Ireland School of Medicine, Dublin, Ireland.; Laboratory of Medical Microbiology, Vaccine and Infectious Disease Institute (VAXINFECTIO), University of Antwerp, Antwerp; Laboratory of Clinical Microbiology, Antwerp University Hospital, Edegem, Belgium.; Julius Center for Health Sciences and Primary Care, University Medical Center Utrecht, Utrecht University, Utrecht, the Netherlands.; Department of Primary Care Health Sciences, University of Oxford, Radcliffe Observatory Quarter, Oxford, UK.

**Keywords:** coronavirus, COVID-19, Europe, oseltamivir, primary care, randomised controlled trial

## Abstract

**Background:**

Patients infected with the novel coronavirus (SARS-CoV-2) are being treated empirically with oseltamivir, but there is little evidence from randomised controlled trials to support the treatment of coronavirus infections with oseltamivir.

**Aim:**

To determine whether adding oseltamivir to usual care reduces time to recovery in symptomatic patients who have tested positive for coronavirus (not including SARS-CoV-2).

**Design and setting:**

Exploratory analysis of data from an open-label, pragmatic, randomised controlled trial during three influenza seasons, from 2016 to 2018, in primary care research networks, in 15 European countries.

**Method:**

Patients aged ≥1 year presenting to primary care with influenza-like illness (ILI), and who tested positive for coronavirus (not including SARS-CoV-2), were randomised to usual care or usual care plus oseltamivir. The primary outcome was time to recovery defined as a return to usual activities, with minor or absent fever, headache, and muscle ache.

**Results:**

Coronaviruses (CoV-229E, CoV-OC43, CoV-KU1 and CoV-NL63) were identified in 308 (9%) out of 3266 randomised participants in the trial; 153 of these were allocated to usual care and 155 to usual care plus oseltamivir; the primary outcome was ascertained in 136 and 147 participants, respectively. The median time to recovery was shorter in patients randomised to oseltamivir: 4 days (interquartile range [IQR] 3–6) versus 5 days (IQR 3–8; hazard ratio 1.31; 95% confidence interval = 1.03 to 1.66; *P* = 0.026).

**Conclusion:**

Primary care patients with ILI testing positive for coronavirus (not including SARS-CoV-2) recovered sooner when oseltamivir was added to usual care compared with usual care alone. This may be of relevance to the primary care management of COVID-19.

## INTRODUCTION

Patients infected with the novel coronavirus, SARS-CoV-2, and suffering from COVID-19 are currently being treated with drug combinations that include oseltamivir.^1^ The authors had previously found that adding oseltamivir to usual primary care for influenza-like illness (ILI) accelerates recovery by about 1 day in those with ILI, and longer in those with key risk factors in the ALIC^4^E study (a randomised controlled trial of clinical and cost effectiveness in primary care).^2^ This effect did not appear to be mediated by influenza virus status as determined by polymerase chain reaction (PCR) results from nasopharyngeal swabs. Outcomes for patients found positive for coronavirus (not including SARS-CoV-2) had not been analysed separately. Given the evolving pandemic, this study set out to conduct a post-hoc exploratory analysis of the open-label, pragmatic, ALIC^4^E trial data to explore whether adding oseltamivir to usual primary care for patients with ILI who have tested positive for coronavirus (not including SARS-CoV-2) is effective in reducing time to recovery.

## METHOD

### Study design

This was a post-hoc exploratory analysis of data from the ALIC^4^E trial, an open-label, pragmatic, randomised controlled trial, previously described in full.^2^^,^^3^

### Setting and patients

Patients aged ≥1 year presenting to primary care with ILI during three seasonal influenza seasons (15 January 2016 to 12 April 2018) in 15 European countries, randomised in the ALIC^4^E trial and infected with coronavirus (not including SARS-CoV-2) were eligible for this study. ILI was defined as a sudden onset of self-reported fever, with ≥1 respiratory symptom (cough, sore throat, running or congested nose) and one systemic symptom (headache, muscle ache, sweats or chills, or tiredness), with symptom duration of ≤72 hours during a seasonal influenza epidemic.^4^ Coronavirus infection was confirmed using the Fast Track Diagnostics Respiratory Pathogens 21 plus real-time PCR assay on baseline swabs.^5^ An oropharyngeal and nasal swab (COPAN) were taken from those aged <16 years and a nasopharyngeal swab (COPAN) from those aged ≥16 years. PCR results were not available for clinicians to inform management.

**Table:** How this fits in

Patients with COVID-19 are being treated with drug combinations that include oseltamivir. Evidence from randomised controlled trials for oseltamivir therapy is limited. This study, from 2016 to 2018, found that primary care patients with symptomatic coronavirus infection (not including SARS-CoV-2) recovered sooner when oseltamivir was added to usual care. Therefore, oseltamivir might be considered for the primary care management of (suspected) COVID-19.

### Study randomisation

Participants were randomised at the point of care using a remote online electronic data capture system, with a 1:1 ratio between the two arms.

### Intervention

Participants were randomised to either usual primary care or usual primary care plus oseltamivir. Adults and children weighing >40 kg, who were randomised to the intervention and able to swallow capsules, were given 75 mg oral oseltamivir twice daily for 5 days. For those aged <13 years, oseltamivir was given in oral suspension, according to weight: 30 mg for those weighing 10–15 kg; 45 mg for those weighing >15–23 kg; 60 mg for those weighing >23–40 kg; and 75 mg for those weighing >40 kg.

### Procedures

A baseline case report form was completed covering overall clinician-rated ILI severity, duration of symptoms, comorbidity, temperature, pulse, individual ILI symptom patient-reported severities, and usual care advice (registered by clinician).

Patients were asked to complete a symptom diary for 14 days in order to indicate when they had returned to their usual daily activities and to evaluate fever, running/congested nose, sore throat, headache, cough, shortness of breath, muscle ache, sweats/chills, diarrhoea, nausea/vomiting, abdominal pain, low energy/tiredness, sleeplessness, dizziness, and feeling generally unwell as ‘no’, ‘minor’, ‘moderate’, or ‘major’ problem. These were supplemented with child-specific questions so that the Canadian Acute Respiratory Illness Flu Scale was completed for children aged <13 years.^6^ Patients were contacted via telephone after 2–4 days, 14–28 days, and 28 days to support study participation, diary completion, monitor intervention adherence, and to ascertain a minimal outcome data set.

### Outcome measures

The primary outcome was patient-reported time to recovery, defined as having ‘returned to usual daily activity’, and ‘fever’, ‘headache’, and ‘muscle ache’ rated as ‘minor’ or not problematic. For non-verbal children, ‘clinginess’ replaced ‘headache’ and ‘muscle ache’ when both were unanswered.^3^ Where diary data were unavailable, data from the 14–28 days telephone call were used, and if that was unavailable, data from the telephone call after 28 days were used. Where data were incomplete, participants were censored at their last contact date or at 28 days.

### Statistical analysis

Characteristics of the participants with coronavirus infection in the two study arms are presented. For this exploratory data analysis, the authors produced the Kaplan– Meier survival curves for each treatment group and estimated the hazard ratio (HR), 95% confidence interval (CI), and associated *P*-value, comparing treatment groups with a Cox proportional hazard regression model. The analysis was performed on the intention-to-treat (ITT) population, which included all randomised patients in the arm they were assigned regardless of treatment received. Missing data were not imputed.

## RESULTS

**Figure 1.**
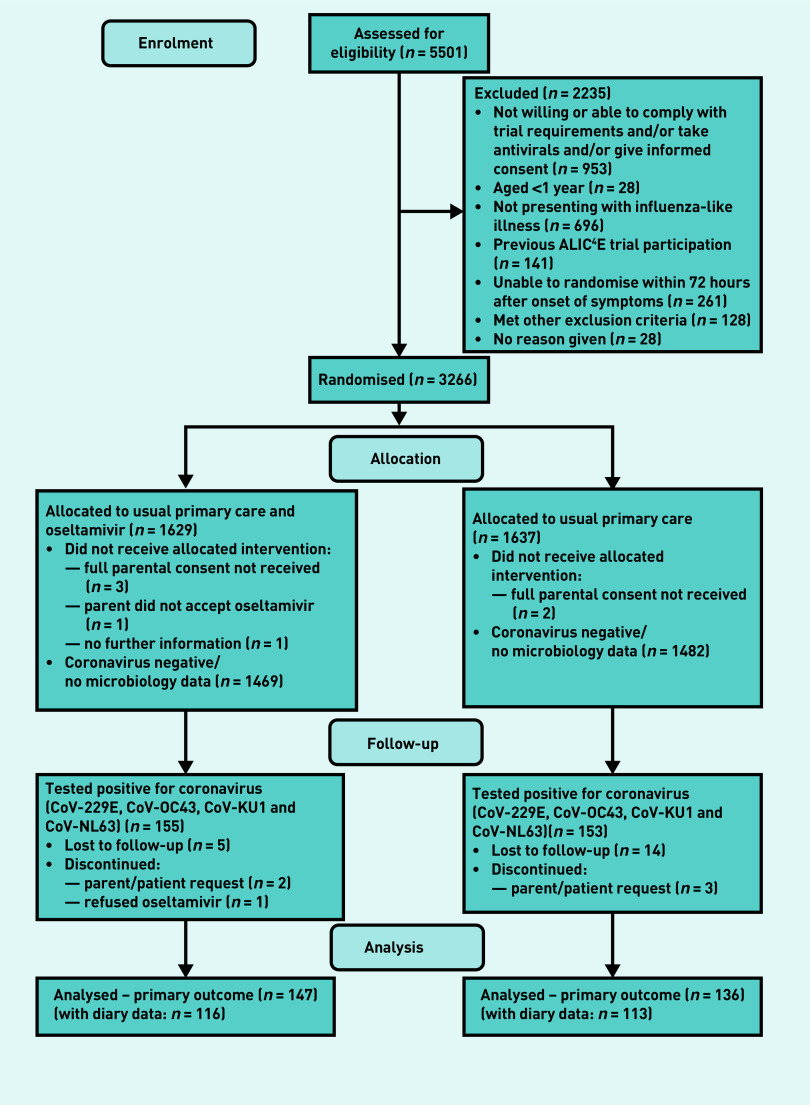
***Flow of patients in the ALIC^4^E trial and of those who tested positive for coronavirus (not including SARS-CoV-2).***

Coronaviruses (CoV-229E, CoV-OC43, CoV-KU1 and CoV-NL63, which are known pathogens in humans) were identified in 308 (9%) of 3266 randomised participants from 21 networks covering 209 primary care practices in 15 European countries over three consecutive influenza seasons. Of these identified cases, 130 were male (42%) and 17 were aged >65 years (6%); 153 were randomised to usual care and 155 were randomised to usual care plus oseltamivir. The primary outcome was ascertained in 136 (89%) and 147 (95%) participants, respectively (Figure 1). Demographic and clinical characteristics were similar between the randomisation groups (Table 1). The 25 patients who did not provide primary outcome data were more often male, aged <12 years, 20 (80%) more often included in the final season, and more often had a chronic respiratory condition (see Supplementary Table S1).

**Table 1.** Baseline demographic and clinical characteristics by treatment group

**Characteristics**	**Usual care, *n* (%), *N* = 153**	**Usual care plus oseltamivir, *n* (%), *N* = 155**
**Demographics**		
Sex, male	65 (42)	65 (42)
Age, years		
<12	14 (9)	15 (10)
12–65	130 (85)	132 (85)
>65	9 (6)	8 (5)

**Comorbidity**		
Heart disease	6 (4)	9 (6)
Diabetes	6 (4)	7 (5)
Chronic respiratory condition	12 (8)	11 (7)
Hepatic, hematologic, neurological, neurodevelopmental condition	2 (1)	0 (0)
Stroke/transient ischaemic attack	1 (1)	1 (1)
Overnight hospital stay in preceding year	5 (3)	4 (3)

**Influenza season**		
2015–2016	32 (21)	29 (19)
2016–2017	68 (44)	63 (41)
2017–2018	53 (35)	63 (41)

**Figure 2.**
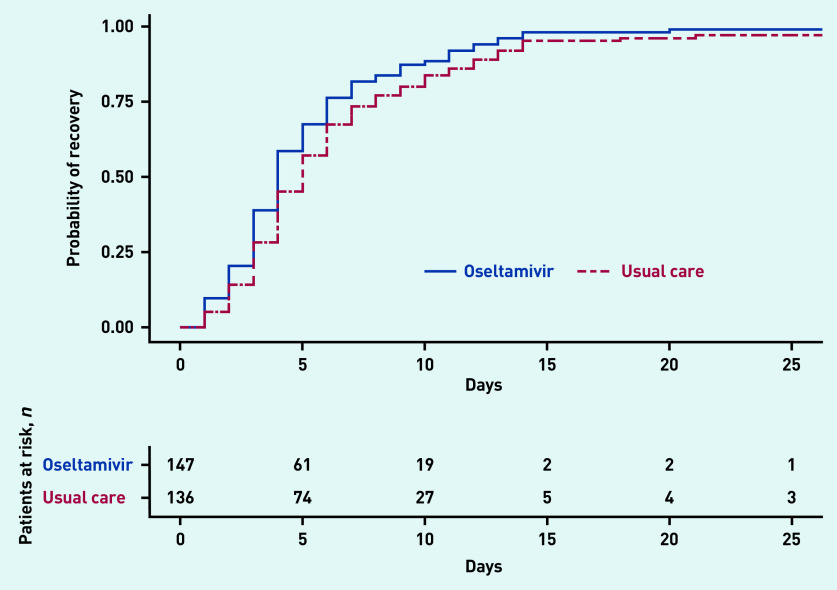
**Kaplan–Meier curve of time to recovery.**

The Kaplan–Meier plots for time to recovery show faster recovery in patients treated with oseltamivir (Figure 2), with a median of 5 (interquartile range [IQR] 3–8) days for participants randomised to usual care versus 4 days (IQR 3–6) in participants randomised to usual care plus oseltamivir. The mean number of days to recovery for patients was 6.35 days (standard deviation [SD] = 4.93) in the usual care group and 5.20 (SD = 3.93) days in the oseltamivir group. The HR was 1.31 (95% CI = 1.03 to 1.66, *P* = 0.026) favouring oseltamivir.

In the usual care group, 54 patients contacted their GP (70 contacts) versus 57 patients in the oseltamivir group (72 contacts) in the first week after inclusion. In the second week after inclusion, 17 patients in the usual care group contacted their GP (21 contacts) versus 14 patients in the oseltamivir group (16 contacts) (data not shown). In the usual care group, seven patients visited the hospital in the 4 weeks after inclusion, of which one stayed overnight, two had an X-ray, with one confirmed pneumonia. In the oseltamivir group, one patient visited the hospital, none stayed overnight, and none had an X-ray (data not shown).

## DISCUSSION

### Summary

This exploratory analysis of the ALIC^4^E trial data from 2016 to 2018 suggests that primary care patients with ILI, who tested positive for coronavirus (not including SARS-CoV-2) and received usual care, returned to their usual activities with relevant residual symptoms, minor or absent, in a median of 5 days (mean 6.35 days). Patients also receiving oseltamivir returned about 1 day sooner.

### Strengths and limitations

The present pragmatic, open trial design did not allow identification of mechanisms of action, or a measure of how much of the observed effect can be attributed specifically to oseltamivir or other possible effects, but allows the observed results to likely reflect real world effects in primary care.^7^^,^^8^ It should be noted that this was a primary care study and that the findings cannot be extrapolated to more severely ill and/or hospitalised patients. In addition, though unlikely, SARS-CoV-2 may respond differently to oseltamivir.

### Comparison with existing literature

This study’s findings are consistent with other studies showing benefit of oseltamivir in all patients with ILI,^2^ and with previous placebo-controlled evidence for adults and children with ILI, irrespective of infection by influenza or another virus.^9^^–^^12^ Previously published possible explanations include that oseltamivir’s mode of action may include generalised non-specific mechanisms, and/or an action on a wider range of viruses,^10^ or, that a placebo effect was found in the present study. However, in the ALIC^4^E trial there was no evidence of a differential relative benefit in subgroups, such as those with lower illness severity where systematic reviews suggest a more marked placebo response.^13^ In addition, the ALIC^4^E trial’s overall estimate of benefit is similar to effects found in placebo-controlled trials.

### Implications for research and practice

Secondary analysis of data from the placebo-controlled trials of oseltamivir in patients with ILI not caused by influenza viruses, for example by coronaviruses, and new placebo-controlled trials in patients with COVID-19 could help elucidate a causal effect for its benefit in those patients. Meanwhile, adding oseltamivir to usual primary care appears to accelerate recovery by about 1 day in patients with ILI who test positive for coronavirus (not including SARS-CoV-2), and, though the present study has not proven that SARS-CoV-2 responds to oseltamivir, this drug could be considered for the management of primary care patients with (suspected) COVID-19.
